# The Dimensions of Transformational Leadership and Its Organizational Effects in Public Universities in Saudi Arabia: A Systematic Review

**DOI:** 10.3389/fpsyg.2021.682092

**Published:** 2021-11-10

**Authors:** Ghuzayyil Saad Alessa

**Affiliations:** Department of Public Administration, College of Business Administration, King Saud University, Riyadh, Saudi Arabia

**Keywords:** transformational leadership, the effects of transformational leadership, Saudi public universities, academic leadership, organizational sustainability

## Abstract

Organizations are dynamic entities, such that they are constantly developing and changing. As such, these entities require leadership capable of managing transformations. Transformational leadership is an effective leadership model that focuses on adapting to existing environmental circumstances whether through internal information, human and monetary resources, or different external variables. This study aims to highlight the characteristics of transformational leadership and their effects on the public universities in Saudi particularly, this study enlightens the urgent need to test the behavioral intentions in the public universities in Saudi Arabia. To achieve this goal, many major databases were used. The period of the study was set from 2011 to 2020 as the topic of the study is the current transformational leadership within the public universities in Saudi Arabia. There was a total of 47,400,000 studies on transformational leadership on Google, however, only 22 studies were reviewed which were entirely conducted in Saudi Arabian Universities. The results demonstrated that in the public universities in Saudi, transformational leadership is practiced across four dimensions: ideal effect, inspirational motivation, intellectual stimulation, and individual consideration. The documentary research approach was used to review the most recent aspects of administrative literature on the theory of transformational leadership and its organizational outcomes. In addition, NVIVO 11 was used to make a thematic analysis of the linkages between transformational leadership and organizational outcomes: organizational commitment, knowledge management practices, morale, employee empowerment, level of job satisfaction, administrative creativity, organizational citizenship behavior, and the level of transformation toward quality and job enrichment. Therefore, behavioral tendencies such as organizational commitment, empowerment, job satisfaction, and knowledge management practices have been studied more interestingly and critically. Furthermore, these behavioral aspects need to be explored more in public universities in Saudi Arabia.

## Background

Organizational success and the effectiveness in achieving goals based on a clear mission and vision usually correspond to leadership approaches that target raising service levels, the introduction of modern organizational development trends, and adaptation to internal and external environments. Accordingly, choosing administrative leaders who will take on the responsibility of making change and bearing its risks is of high importance to organizations. Transformational leadership involves factors, characteristics, and tasks that enable leaders to face current and future challenges and effectively manage the organizational changes resulting from continuous external changes. As such, this study will address the topic of transformational leadership with an emphasis on the characteristics of transformational leadership and its effects on the public universities in Saudi. The researcher reviewed the articles which were ultimately done within the universities in Saudi Arabia. The number of research studies on Google was 47,400,000 which were conducted globally, however, this case was completely conducted with Saudi Arabian universities so, only 22 studies, purely based on transformational leadership and its possible outcomes, were selected for the systematic review.

### The Roots of Transformational Leadership

Transformational leadership theory is one of the oldest psychological and methodological approaches used in understanding and interpreting leadership itself. It is described by Burns (1978) as a process related to the internal relations and values in which a leader influences others and adapts their behavior to meet challenges, ultimately enabling them to participate in the process of organizational change (Tengi et al., 2017: 79). The emergence of the transformational leadership theory can be traced back to Burns (1978), who was the first to address: “…the distinction between transactional leaders, who attempt to satisfy the current needs of followers by focusing attention on exchanges, and transformational leaders, who attempt to raise the needs of followers and promote dramatic changes of individuals, groups, and organizations” (Yammarino et al., 1993: 81–82). Burns (1978) suggested that leadership, in addition to the ability of the leader to influence followers, includes the ability to motivate others and develop their moral values to help bring about change in the behaviors, attitudes, values, and expectations of the followers, and then change the behavior of the organization as a whole (Tengi et al., 2017:794). Transformational leaders generally tend to show four main characteristics: charisma, inspirational leadership, intellectual stimulation, and the consideration of the needs of followers (Dubinsky et al., 1995:317).

By the mid-eighties, the concept of transformational leadership received significant attention from scholars in the field of management, especially when many organizations realized the need for major changes in the ways in which business is carried out as a means of addressing the changes in the environment. Bass (1985) reviewed Burns' theory on the effect of transformational leadership and proposed a more detailed model for describing the transformational processes in organizations, where the transformational leader was defined as the one who transforms a vision into reality by motivating the followers to substitute their personal interests for the interest of the group. The focus in this study is on the characteristics of a transformational leader in transforming followers by meeting their needs, making them more knowledgeable about the importance and value of the outcomes of their jobs, and persuading them to sacrifice their individual interests for the interests of the organization. As a result, the followers express an increase in job satisfaction levels, respect toward leaders, and motivation in task execution (Tengi et al., 2017:794).

### The Concept of Transformational Leadership

Dubinsky et al. (1995) explained that the concept of transformational leadership is a reflection of several characteristics found in leaders, such as the acknowledgment of future needs and issues, handling of long-term problems and opportunities, holistic examination of internal and external organizational factors, handling of organizational issues from a broad perspective, elevation of follower awareness regarding the importance and value of specific job outcomes, ability to motivate employees to substitute their personal interests for those of the organization, and ability to influence followers to change their needs to higher-order concerns. In the same context, a transformational leader is a leader who has influence and can interact directly with followers to change various aspects of an organization through vision, action, and impact. Leadership involves the attitude and behavior of a person to influence a team to be able to work together more efficiently and effectively to achieve a required level of productivity (Tengi et al., 2017:792).

It is essentially the process through which leaders and followers are committed to achieving goals within a framework of vision, shared values, and mutual trust. In this process, the leaders encourage the followers to pursue personal development and adaptation skills, and as a result, the leaders and followers raise each other to the highest level of motivation. It is through this focus on human behavior and motivation that transformational leadership has a significant transformational effect on leaders and followers, as well as on the performance and development of the organization as a whole.

### The Dimensions of Transformational Leadership

Both Savovic (2017) and Wood (2019) asserted that one of the most important tasks of transformational leadership involves enhancing the participation between leaders and followers in terms of motivation and values, while also enhancing the awareness of the followers on existing problems and providing support, encouragement, and developmental experience. This requires that leaders focus on developing the abilities of the followers to creatively find solutions to problems, providing them with a blueprint for the future that inspires them and provides them with the support they need to face the challenges of change, ultimately increasing their commitment to efficient task implementation. Ultimately, transformational leaders inspire changes in the attitudes and core values of their followers to foster its alignment with the organizational vision.

According to Burns, transformational leadership can be seen when the leaders and followers push one another to higher levels of morals and motivation. Through the power of their vision and personality, transformational leaders can inspire followers to change their hopes, perceptions, and motivations, and work toward common goals. Transformational leaders must be able to define and communicate the vision of an organization, while subordinates must acknowledge the credibility of their leaders as transformational leaders who are charismatic and play a central and strategic role in helping the organization achieve its goals. Transformational leaders must also be able to balance their future visions with those of their subordinates while attributing greater importance to the needs of their subordinates than what may exist at present. Furthermore, transformational leaders must be able to persuade their subordinates to carry out tasks beyond their interests for the greater good of the organization (Gunawan, 2020).

Therefore, the role of administrative leaders in managing change is of great importance because organizations are dynamic entities undergoing rapid development, and are therefore in need of leadership that is capable of managing transformations. Hence, the administrative leader who continuously strives to improve their performance and personal skills and simultaneously deals with the rapidly-changing modern global variables can be identified as a transformational leader. The administrative leader who wants to achieve extraordinary things at a level that exceeds expectations must be updated on the new trends in management and technology, while also acquiring new skills that help anticipate and internalize future changes.

### Organizational Effects of Transformational Leadership

The transformational leadership approach contributes to keeping organizations abreast of all the surrounding changes, as their leaders and members have a clear vision of the future that they are working hard to achieve. Many studies on transformational leadership indicate a positive correlation between transformational leadership and positive organizational outcomes. Therefore, this systematic review significantly analyzes the individual characteristics and effects of transformational leadership on organizational outcomes such as: It is clear from each perspective of the dimensions of transformational leadership that it makes a significant contribution to the actual performance of academic educational institutions. This is because all the dimensions possess motivational and cognitive abilities that are considered necessary for the development and evaluation of academic performance in academic institutions (Al Gabri, 2018); According to Al Amiri (2002), transformational leadership in public institutions in Saudi promotes a high level of staff opinions and results in higher academic performances; it has the best practices in developing and generating novel ideas that facilitate the process of knowledge sharing in terms of knowledge management (Al Madhahaji, 2017); it is the best practice to satisfy employees at work, and as a result, the employees go above and beyond to satisfy their employers (Al Madhahaji, 2017). According to Al Miman (2013), transformational leadership has an influential effect on organizational creativity in terms of knowledge sharing practices, developing novel ideas, and doing best practices and processes in private and public higher institutions in Riyadh (Al Rashidi, 2017); it enables corporate social responsibility among the faculty members of King Saud University (Al Regeb, 2017); it helps to empower knowledge management system in public universities in Saudi (Al Saleh, 2019); and it is the ultimate antecedent of both organizational commitment and organizational citizenship behavior (Al Ubiri, 2016) because it enhances faculty commitment and citizenship behaviors among the faculty members of public higher institutions.

#### The Effect of Transformational Leadership on Employee Performance

Some studies, such as the empirical study of Buil et al. (2019) on the impact of transformational leadership on employee performance, have investigated the effect of transformational leadership on employee performance and its related aspects. Measures were used to assess transformational leadership among 323 employees. They included: organizational identification, work engagement, job performance, and proactive personality. The findings supported a link between the transformational leadership approach and the level of job performance. They stated that “both identification and engagement, as mediator variables, govern the underlying mechanism of the relationships between transformational leaders and their followers' behaviors.” Their findings suggest that the use of the transformational leadership approach can predict job performances because those leaders “motivate their followers to identify with their organizations, which, in turn, increases their level of engagement.”

Wood (2019) compared three leadership theories, namely, transformational leadership, situational leadership, and reciprocity, to examine the suitability of these theories to the environment of contemporary business and he found that transformation leaders were undoubtedly more capable of improving the performance of an organization by empowering employees and enabling change. Similarly, the lack of success of institutions was related to the absence of effective leadership that promotes the necessary behaviors for achieving organizational goals. Thus, it was found that although transformational leadership was proposed many decades ago, it is still considered the most appropriate for present times due to its ability to facilitate organizational innovation and learning while promoting a shared and inspiring vision for the future.

Savovic (2017) also studied the impact of transformational leadership on job performance, considering the variables of organizational change, growth, integration, and expansion of activities. The previous study was based on a sample of 344 employees in a Serbian company. The results showed that transformational leadership, in all its dimensions, has a positive effect on job performance during the change process, as it leads to a reduction in uncertainty, making employees more accepting, and increasing their commitment to the organization. They found that transformational leaders influence job performance positively through their charismatic performance, individual support for employees through guidance and education, provision of incentives, inspiration, and optimism, as well as encouraging their employees to be innovative and find new ways to solve problems. Additionally, transformation leadership encourages the subordinates to be more productive to the firm regardless of whether their appraisal is measured by unit level or firm level (Buil et al., 2019). Similarly, Yang et al. (2019) supported the influence of transformational leadership on the task performance and contextual performance of employees in the Chinese context. The study also indicated that one of the main factors of the growth, diversification, and access to new markets of a company is the use of transformational leadership during the process of change, especially in the critical stages of adaptation and trying to achieve difficult goals. In an earlier study, Yammarino et al. (1993) developed a model of transformational leadership to investigate its impact on the level of performance. The model was tested in a longitudinal study on a sample of 186 officers in the US Navy who were graduates of the Naval Academy in the United States. The study found a positive relationship between academic and military performance and transformational leadership, which generally reflects positively on the level of job performance in the fleet.

Tengi et al. (2017) also conducted a review on the school leaders in Malaysia based on the theory of transformational leadership, which was widely applied in the Malaysian school leadership system. The study showed that transformational leadership increases organizational effectiveness and that the transformational leader is an influential leader who can interact directly with followers. They asserted that transformational leaders have the ability to inspire organizational change through vision, action, and influence. This style of leadership is about the attitude and behavior of a leader, and how it influences the followers to work together efficiently and effectively to achieve productivity. It was found that the shift to a transformational leadership style plays an important role in increasing the employee performance of school leaders.

Gareth and Gill (2012) investigated transformational leadership across the hierarchical levels in manufacturing organizations in the United Kingdom (UK). The study sample was 367 managers from all organizational levels (upper, middle, and low) working in 38 UK organizations in the industry sector. One of the most prominent results of the study was that the transformational leaders were the most productive and that this leadership style was equally effective across all the hierarchical levels in the organizations that were studied. However, transformational leadership was found to be more widespread at the higher managerial levels.

#### The Behavioral Effects of Transformational Leadership

Real leaders positively influence the behavior of their followers because these leaders provide support for the self-determination of their followers, thus, they are more effective in enhancing the self-motivation of their workers, which in turn leads to an increase in the job satisfaction of their followers (Penger and Cerne, 2014). Some studies investigated the behavioral effects of transformational leadership, such as Purnomo and Novalia (2018). They were interested in the relationship between transformational leadership and job satisfaction in the context of organizational commitment. Their study targeted a sample of 70 Indonesian Airlines employees and found that transformational leadership plays a critical role in influencing the organizational commitment of the employees. The findings of these studies, directly and indirectly, indicated that leaders may increase the performance of the employees and stimulate the creativity of their followers. Whereas, these conclusions indicated the effect of leadership on the performance of employees by the moderating role of proactive personality (Buil et al., 2019). Transformational leaders are found to influence organizational commitment by: (a) promoting higher levels of commitment to goal achievement; (b) creating a higher level of personal commitment to the vision, mission, and shared organizational goals on the part of leaders and followers; and (c) motivating employees to work more effectively, leading to higher levels of organizational commitment. Buil et al. (2019) have conducted a study on transformational leadership and its ultimate outcomes. They supported that transformational leadership, directly and indirectly, influences the performance of employees by the mediating role of work engagement. They further supported that work engagement is only possible when the leaders transparently involve themselves in the tasks of their subordinates at the workplace. Transformational leadership was also studied in relation to job satisfaction. Bruch and Walter (2007) investigated the hierarchical effect of transformational leadership and organizational development by surveying 448 managers from several multinational corporations in Sweden. The aforementioned study covered four basic dimensions of transformational leadership: idealized influence, inspirational motivation, intellectual stimulation, and individualized consideration. The study concluded that idealized influence and inspirational motivation occurred more frequently with the upper- rather than mid-level management, and no differences were found in intellectual stimulation and individualized consideration. The results also indicated that idealized influence, inspirational motivation, and intellectual stimulation are more effective in enhancing job satisfaction for the followers at the upper management levels rather than the middle management levels. Individual consideration was effective in both the groups of upper and middle management. The study emphasized the need to encourage the use of transformational leadership approaches at the lower management levels.

Transformational leadership was also studied in relation to organizational health. Thibault et al. (2019) discussed transformational leadership within Occupational Health Psychology, pointing out that it is associated with positive effects on the performance, well-being, and safety of employees. Leaders can help their followers cope with the negative consequences of work stress and create a positive atmosphere, thus, serving as an intervention for improving organizational health. Several factors affecting organizational health and knowledge sharing were identified by Tuan (2013). In his study, a questionnaire was distributed to 635 middle managers working in 127 Vietnamese companies who were selected because they were able to observe the job behaviors displayed by the senior and lower management better. The study findings showed a strong relationship between transformational leadership and organizational health. Transformational leadership was found to be a healthy management approach that activates the “dynamic interaction” and “stimulates change” among the members of the organization, thus strengthening organizational health.

Al Amiri (2002) conducted a study on transformational leadership in public institutions in the Kingdom of Saudi Arabia on a sample of 466 employees. The findings indicate that transformational leadership positively affected many organizational aspects such as job satisfaction and sense of organizational justice of employees, and organizational citizenship behavior. The study found that personal factors such as age, educational qualification, and job experience did not affect the perceptions of employees on transformational leadership. The study emphasized that government agencies should conduct training courses for managers to establish their leadership skills in change and transformational leadership.

#### The Effect of Transformational Leadership on Organizational Change

Many studies, such as that of Gunawan (2020) on the effect of transformational leadership, school culture, and work motivation on school performance and effectiveness, looked at the role of transformational leadership in organizational change. The data sample included 343 teachers in junior high schools in 44 government high schools in Medan City. The results showed that transformational leadership was an effective approach in initiating organizational change and development. Transformational leaders were found to strive to make a difference and take responsibility for organizational transformation. Moreover, the findings showed that transformational leaders involve their followers in achieving the goals and objectives set by the management of the organization. All in all, it was found that transformational leadership had a positive effect on the motivation level of followers and the organizational culture, thus making it an important variable in the context of organizational effectiveness.

Al Qatawenh (2018) investigated the relationship between transformational leadership and change management in Jordan. The study focused on several dimensions, including idealized influence, inspirational motivation, intellectual stimulation, and empowerment. The data was collected from 500 employees in several Jordanian insurance companies. The findings confirmed that the dimensions of transformational leadership were indeed present in the Jordanian insurance companies and that a positive impact could be observed regarding change management. Therefore, transformational leadership behaviors were recommended to be implemented as they will increase the perception of employees to manage change.

On the other hand, Al Qura'an (2015) aimed to identify the impact of transformational leadership on managing organizational change from the perspective of the managers. The subjects were 50 branch managers of Jordan Ahli Bank. The findings showed that transformational leadership affects the management of organizational change. A positive correlation was found between transformational leadership dimensions (ideal influence, inspirational motivation, intellectual stimulation, individual thinking, and empowerment) as independent variables, and structural change, technological change, and organizational change of personnel as dependent variables. There is a positive relationship between the dimensions of transformational leadership (ideal influence, inspirational motivation, intellectual stimulation, and empowerment) and the management of change. This shows that transformational leadership is very effective in developing and changing organizations because transformational leaders work to make a difference and take on the responsibility of creating change.

### Transformational Leadership in Public Universities in Saudi

Universities are one of the foundational elements in building societies and contributing to progress and development. This is accomplished through university leaders who support the effective achievement of the strategic plans, visions, and goals of the university (Al Shammari, 2020:2). The effective achievement of the desired visions and goals requires leaders who have special capabilities that enable them to face contemporary challenges and deal with them efficiently (Alsomaly and Metoly, unpublished:19). Universities are considered one of the most important tributaries for influencing and changing the attitudes and behaviors of employees while urging them toward the achievement of visions and aspirations. This is why universities need conscious leaders equipped with the characteristics of transformational leadership, such as inspirational motivation, intellectual stimulation, ideal influence, and individual considerations to achieve their development plans (Alaklobee, unpublished:422).

The academic and administrative leadership of the universities in Saudi requires the skills to deal with contemporary challenges, future aspirations, and internal/external changes. This must be balanced with the active management of development due to the pivotal role that Saudi Arabian universities play in the achievement of sustainable, comprehensive development, and the 2030 vision of the Kingdom under new university regulations. This necessitates leaders to assume a transformational leadership approach with its four dimensions (idealized influence, inspirational motivation, intellectual stimulation, and individualized consideration), as this style of leadership is associated with the ability to keep up with rapid developments while influencing the behavior of organizational members and improving their capabilities through encouragement, involvement in problem-solving, empowerment, and participation in decision-making.

## Methodology

In this study, the documentary research approach was used to review administrative literature on the theory of transformational leadership, its concepts, historical roots, tasks, and dimensions. The study also reviewed empirical research on the organizational effects of transformational leadership. Finally, a systematic review of the practice of transformational leadership among the academic leaders in the public universities of Saudi was conducted through extensive research on Arabic and English literature in academic online databases. The resources accessed contained a wealth of information databases available on “Google Scholar” and the “Saudi Digital Library,” such as Proquest, Sage Business Cases, Dar Almandoumah, Almanhal, Arabic Book Collection, and e-Marefa. These databases were chosen due to their comprehensive lists of studies on transformational leadership carried out in the Kingdom of Saudi Arabia, thus encompassing the target population of the academic leaders in Saudi Arabian universities.

**Figure 1:**
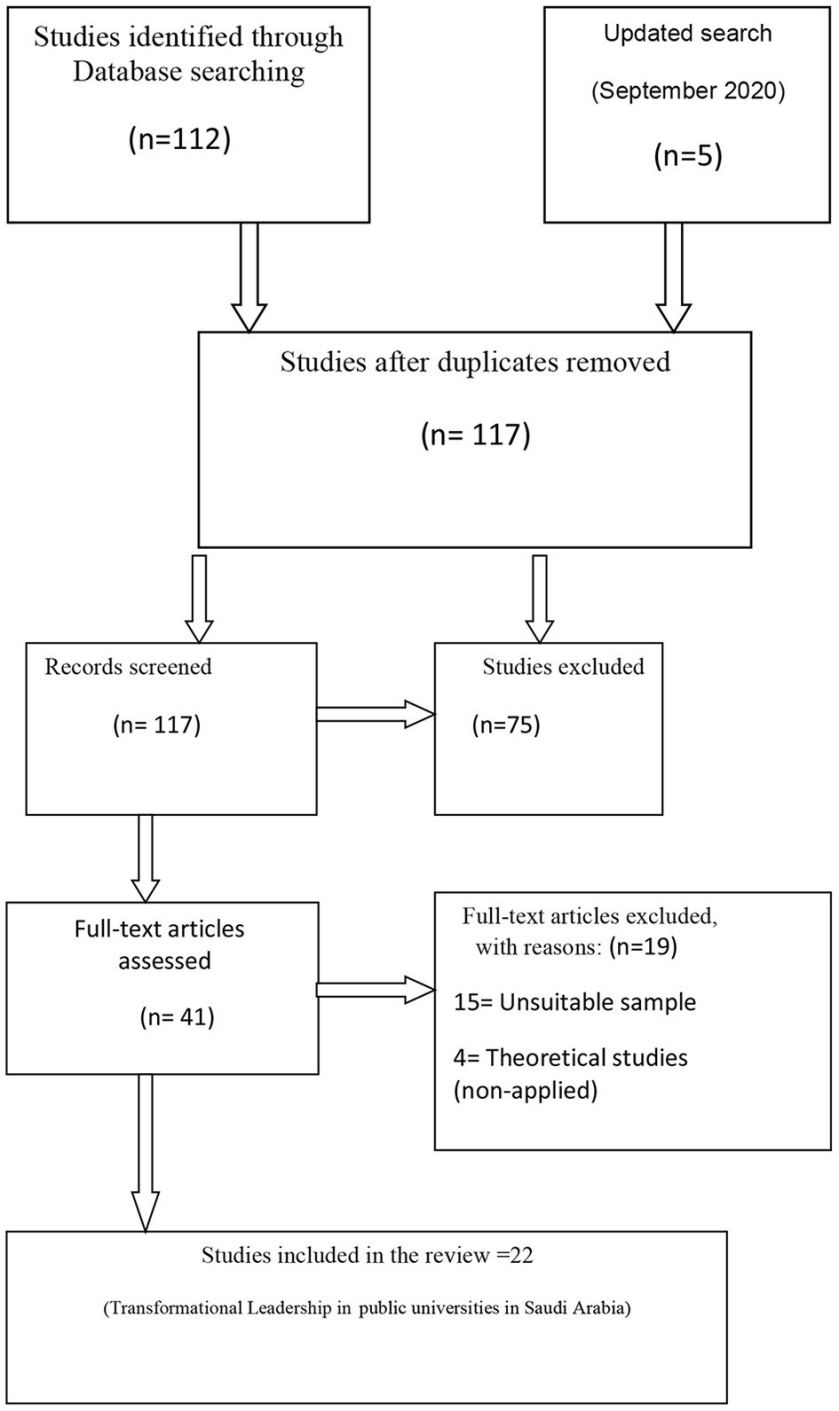
Prisma flow diagram (2011–2020).

The systematic review of transformational leadership and its predicted organizational outcomes has been screened and skimmed by doing a thematic analysis using NVIVO 11 (QSR International, Melbourne, Australia). The contextual and thematic analyses were organized to see the most required organizational outcome in the studies conducted in Saudi Arabian universities between 2011 and 2020. Twenty-two studies on the scope and application of transformational leadership in Saudi Arabian universities were obtained as seen in Figure 1. The extraction of data included: the author, journal, study design, study sample, study measurement tools, measures of validity and reliability, independent and dependent variables, methods of statistical analysis, and study results with respect to the practice of transformational leadership among the academic leaders in Saudi Arabian universities. The regulatory and behavioral implications were also considered.

### A Systematic Review of Literature Findings

In this section of the study, we discuss several recent studies published between 2011 and 2020 that investigated the practice of transformational leadership in Saudi Arabian universities. The goal of this is to explore the extent of the application of transformational leadership, as well as its organizational and behavioral implications.

#### Quantitative Literature Findings

Initially, the studies obtained through the databases were reviewed to ensure that they followed robust scientific methodological procedures. Accordingly, 22 studies were included as shown in Table 1, summarizing the study sample, degree of response, validity and reliability measurements of the study tool, and statistical methods used in the study.

**Table 1:** Characteristics of the literature studies.

**Ref#**	**References**	**Sample**		**Validity**	**Reliability**	**Statistical** **analysis**
		***N***	**RR (%)**			
1	Al Shammari (2020)	166	100	Face validity	α = 0.92	Mean
		King Faisal University				Standard deviation
						*T*-test
						Analysis of variance(ANOVA)
						Scheffe test
2	Al Shammari (2020)	272	77.70	Face and content and	α = 0.995	Mean
		Qassim University		construct		Standard deviation
						*T*-test
						Pearson correlation
3	Al Saleh (2019)	335	100	Face and construct	α = 0.94	Mean
		Umm Al-Qura University, King Faisal University, Princess Nora Bint Abdul Rahman University, Hail University and Jazan University				Standard deviation
						One way ANOVA
						Independent samples test
4	Alaklobee (unpublished)	2019	60	Face validity	α = 0.984	Mean
		University of Shaqraa				Standard deviation
						*T*-test
						Multiple regression
						Analysis of variance(ANOVA)
						Pearson correlation
5	Al Zahrani (2019)	214	61	Content and construct	α = 0.868	Mean
		Albaha University				Standard deviation
						Pearson correlation coefficient
6	Wazrah (2019)	275	100	Face and construct	α = 0.984	Mean
		The				Standard deviation
		University colleges in Wadialdawasir				Pearson correlation
						One way ANOVA
						Tukey test
7	Al Gabri (2018)	34	43	Face and construct	α = 0.93	Mean
		King Saud University				Standard deviation
						One way ANOVA
8	Alsomaly and Metoly (unpublished)	333	83	Face and construct	α = 0.98 3	Mean
		King Abdul-Aziz University				Standard deviation
						Multiple Linear Regression
						Multicollinearity
9	Al Mandil and Shawi (2018)	645	100	Face and construct	α = 0.979	Mean
		King Abdul-Aziz University				Standard deviation
						*T*-test
						Multiple regression
						ANOVA
10	Atoum (2018)	350	100	Construct	α = 0.95	Mean
		King Saud University, Imam Muhammad bin Saud University,				Standard deviation
		The Arab Open University, Al-Faisal University, and Princess University				*T*-test
		Nora, Prince Sattam University, Dar Al Uloom University, and Al-Yamama University				ANOVA Scheffe test
11	Al Rashidi (2017)	243	83	Face and construct	α = 0.98	*T*-test
		Al Jouf University				Analysis of variance
						Regression Analysis
12	Al Sharari (2017)	161	46	Content	α = 0.90	Mean
		Qassim University				Standard deviation
						3 way ANOVA
13	Al Regeb (2017)	246	36	Face and construct	α = 0.85	Mean
		University of Tabuk				Standard deviation
						Analysis of variance(ANOVA)
14	Al Madhahaji (2017)	93	100	Face and construct	–	Mean
		University of Tabuk				Standard deviation
						*T*-test
						Analysis of variance(ANOVA)
						Scheffe test
15	Al Ubiri (2016)	58	72.5	Face and content	α = 0.98	Mean
		King Saud University				Standard deviation
						Pearson correlation
16	Zein Alabdien (unpublished)	357	73.20	Face and construct	α = 0.89	Mean
		Imam Muhammad bin Saud University, and Majmaah University				Standard deviation *T*-test
17	Al Thuwaini (2014)	98	100	Face validity	α = 0.953	Arithmetic Mean
		Jazan University, Al-Jouf University, Tabuk University, Al- Alhudud Alshamalia University, Najran University, Majmaah University- Shaqra University, University of Dammam				Standard deviation
						*T*-test
						One way ANOVA
18	Burqan (2013)	203	58	Face and content	α = 0.97	Mean
		Saudi Arabian Technical Colleges				Standard deviation
						*T*-test
19	Al Subaie (2013)	350	100	Face and content	α = 0.97	Mean
		Taif University				Standard deviation
						Pearson correlation
						One way ANOVA
						Mann-Whitney *U*-test
20	Al Miman (2013)	243	83	Face and construct	α = 0.92	Mean
		King Saud University, Al-Jouf University, Najran University, Al-Baha University				Standard deviation
21	Ayyad (2013)	166	100	Face validity	α = 0.93	Mean
		King Faisal University				Standard deviation
						Pearson correlation
						AMOS
22	Al-Shammari (2015)	272	77.70	Face validity	α = 0.85	Mean
		Qassim University				Standard deviation
						*T*-test
						One way ANOVA

#### Using Likert Scale and Mean Score of Response Rate (Likert Scale)

The arithmetic means and SD were calculated to measure the degree of response of the sample individuals, as well as to gauge their opinions on the paragraphs of transformational leadership dimensions featured in the questionnaires of the studies that were applied to Saudi Arabian universities. With regards to determining the level of transformational leadership practice, a criterion divided into three equal categories was adopted, where the value was calculated by dividing the difference between the upper and lower value of the scale into three degrees and filling the number of levels [(5–1)/3 = 1.33]. The three categories were divided into the low level (1–2.33), medium level (2.34–3.67), and high level (3.68–5). Reviewing the field studies conducted on the public universities in Saudi provides a clear summary of the current evidence on the topic of transformational leadership practices over 10 years. Table 2 shows the synthesis of studies according to a measurement scale of the “level of practice.” The results show that the studies investigated transformational leadership practices and that the level of practices found in the sample on the four dimensions of transformational leadership—idealized influence, inspirational motivation, intellectual stimulation, and individualized consideration—ranged between two levels such as high and medium. This indicates that there is a high level of agreement among the participants from different studies on the level of practice of transformational leadership of academic leaders. In light of the earlier discussion on transformational leadership, its characteristics, and positive effects, this finding suggests that the academic leaders believed that this method fits the organizational work environment in their universities and their improvement objectives to achieve the vision of Saudi Arabian universities on development, effectiveness, quality, and academic excellence.

**Table 2:** The mean score of the opinions of the participants of the studies on the extent to which their managers possess transformational leadership characteristics within the dimensions of idealized influence, inspirational motivation, intellectual stimulation, and individualized consideration.

**Ref#**	**References**	**The mean score of the opinions of the studies' participants on the extent that their managers possess transformational leadership characteristics**		
		**Level of practice scale**		
		**High (3.68–5)**	**Medium (2.34–3.67)**	**Low (1–2.33)**
1	Al Shammari (2020)		3.61	–
2	Al Shammari (2020)	3.76		–
3	Al Saleh (2019)		3.63	–
4	Alaklobee (unpublished)	4.22		–
5	Al Zahrani (2019)		3.04	–
6	Wazrah (2019)	4.02		–
7	Al Gabri (2018)		3.21	–
8	Alsomaly and Metoly (unpublished)	3.85		–
9	Al Mandil and Shawi (2018)	3.76		–
11	Al Rashidi (2017)		3.61	–
12	Al Sharari (2017)	4.18		–
14	Al Madhahaji (2017)		3.62	–
15	Al Ubiri (2016)	3.83		–
16	Zein Alabdien (unpublished)	4.03		–
17	Al Thuwaini (2014)	3.73		–
18	Burqan (2013)		3.64	–
19	Al Subaie (2013)	3.98		–
20	Al Miman (2013)	3.89		–
21	Ayyad (2013)		3.11	–
22	Al-Shammari (2015)		2.64	–

#### Correlation Coefficient Between Transformational Leadership and Organizational Outcomes

Table 3 shows the results of the review of studies regarding the organizational and behavioral effects of the practice of transformational leadership by leaders in Saudi Arabian universities in its various dimensions: idealized influence, inspirational motivation, intellectual stimulation, and individualized consideration. The results indicate that there is a statistically significant positive correlation between the practice of transformational leadership and employee empowerment, employee creativity, managerial creativity, knowledge consultation, and the practice of knowledge management (obtaining knowledge, sharing knowledge, organizational learning), organizational commitment, job enrichment, morale, the level of job satisfaction, organizational citizenship behavior, orientation toward change to achieve the 2030 vision of the Kingdom, and the level of transformation toward quality. These results, which indicate that the practice of transformational administrative leadership in Saudi Arabian universities has positive effects, are similar to the previous studies that have been mentioned in the review of the literature.

**Table 3:** Linkages between transformational leadership and organizational outcomes.

	**The relationship of transformational leadership**	**Pearson correlation**
Administration creativity	A statistically significant correlation between the practice of transformational leadership and organizational creativity (Al Rashidi, 2017)	0.91
	A statistically significant correlation between the practice of transformational leadership and managerial creativity (Atoum, 2018)	0.875
	A statistically significant correlation between transformational leadership and employee creativity (Alsomaly and Metoly, unpublished)	0.857
	A statistically significant correlation between the practice of transformational leadership and managerial creativity (Ayyad, 2013)	0.710
Employee empowerment	A statistically significant correlation between transformational leadership and employee empowerment (Alsomaly and Metoly, unpublished)	0.865
Knowledge management practices	A statistically significant correlation between the practice of transformational leadership and the behavior of knowledge consultation (Al Zahrani, 2019)	0.732
	A statistically significant correlation between the practice of transformational leadership and the practice of knowledge management (obtaining knowledge, sharing knowledge, organizational learning) (Al Madhahaji, 2017)	0.47
Job enrichment	A statistically significant correlation between the practice of transformational leadership and job enrichment (Ayyad, 2013)	0.65
Organizational commitment	A statistically significant correlation between transformational leadership and organizational commitment (Al Ubiri, 2016)	0.569
Morale	A statistically significant correlation between transformational leadership and morale (Al Shammari, 2020)	0.903
Job Satisfaction	A statistically significant correlation between the practice of transformational leadership and the level of job satisfaction (Al Mandil and Shawi, 2018)	0.89
	A statistically significant correlation between transformational leadership and job satisfaction (Al Miman, 2013)	0.733
Organizational citizenship behavior	A statistically significant correlation between transformational leadership and organizational citizenship behavior (Al Ubiri, 2016)	0.536
	A statistically significant correlation between transformational leadership and the level of change toward quality (Al Subaie, 2013)	0.78
	Transformational leadership characteristics influence organizational change to achieve the Kingdom's Vision 2030 (Alaklobee, unpublished)	0.627

### Qualitative (Thematic and Contextual) Findings Using NVIVO

After screening the systematic review of the literature studies, this study extracted the thematic linkages of transformational leadership with organizational outcomes. The thematic linkages have been found and extracted from the literature studies in Saudi Arabian universities. The next step was organized to develop different “nodes” from the themes of the linkages between transformational leadership and organizational outcomes. Therefore, the present research followed the step by step procedure of NVIVO data analysis as suggested by Alabri et al. (unpublished) in the following circumstances:

1. Converting audio interviews into manuscripts.
2. Unorganized transcription converted into organized manuscripts.
3. Data coding.
4. Generating “themes” and developing “Nodes.”
5. Generating “treemaps” and “charts.”
6. Applying contextual queries:

- *A text search query for* **“*word tree”***
- *Word frequency query for* **“*tag clouds”***
- *Coding queries for* **“*textual data codes in each theme”***

**Figure 2:**
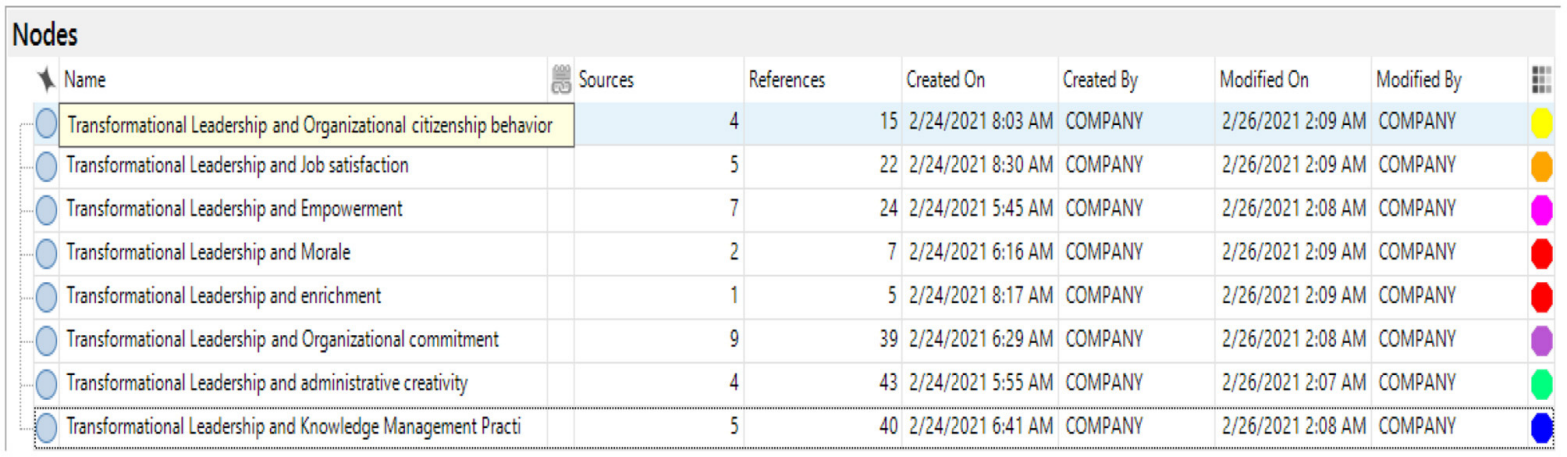
Nodes of contextual linkages.

Figure 2 shows the coding of both the (1) source (S) and (2) reference (R) against generating each theme. The S and R are explained below:

- **Source (S):** It demonstrates the numbers (frequency) of the participants who provided information about a particular theme (factor).
- **Reference (R):** It demonstrates the total number of themes coded in a particular factor about a particular participant. The total number of R is greater than the total number of S because sometimes, one participant talks about a particular theme twice. It means that the present study codes the participant discussion on one theme twice as R and the participant as S.

Figure 2 presents the coding nodes of the linkages between transformational leadership and organizational outcomes. The links between transformational leadership and different organizational outcomes have been identified in literature studies in the public sector universities in Saudi Arabia. For example, the total source of the coding was S = 22 research articles in public Universities. Additionally, the references of the coding were R = 195. The nodes of the linkage (S = 4, R = 15) for transformational leadership and organizational citizenship (S = 5, R = 22), for transformational leadership and job satisfaction (S = 7, R = 24), for transformational leadership and empowerment (S = 2, R = 7), for transformational leadership and moral (S = 1, R = 5), for transformational leadership and job enrichment (S = 9, R = 39), for transformational leadership and organizational commitment (S = 4, R = 43), administrative creativity and (S = 5, R = 40) for transformational leadership and knowledge management practices. This shows that most of the studies in Saudi Arabian universities had been conducted to assess the organizational commitment (emotional attachment) of the professors in these Universities.

### Hierarchy Chart

**Figure 3:**
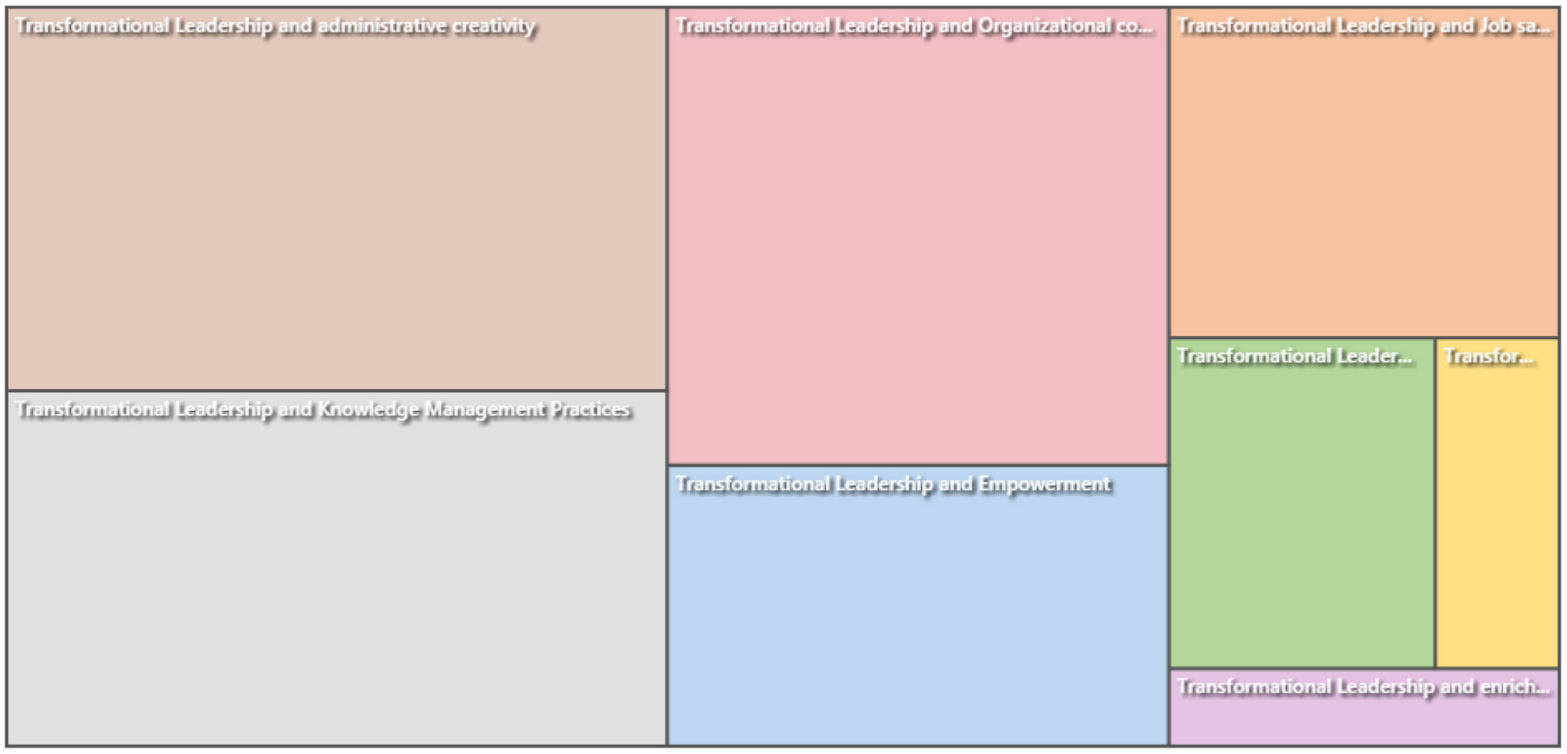
Hierarchy chart.

The hierarchy charts help the readers to visualize the coding patterns and see the values attributing to the cases and sources. Hierarchy charts are proven to be beneficial when the readers aim to explore the views on data and show different opinions. There are two types of charts including hierarchy charts and treemaps. A treemap is used to analyze hierarchies and compare them according to their sizes in data aspects. In addition, it is easier to compare with the rectangular shape of the hierarchy chart than curved shapes. Therefore, in Figure 3, the readers can see and finally analyze that the literature studies that mostly shed light on the linkages between transformational leadership and administrative creativity in the public universities of Saudi Arabia. Second, the researchers focused on the knowledge management practices of the transformational leaders in the public universities of Saudi Arabia. Third, the researchers explored the impact of transformational leadership on organizational commitment. Fourth, the researchers focused on individual empowerment to lead the University culture into the most advanced. Fifth, job satisfaction was assessed by hiring transformational leaders in public universities. Job enrichment was the factor that the researchers focused on the least in the public universities of Saudi Arabia.

### Treemap

**Figure 4:**
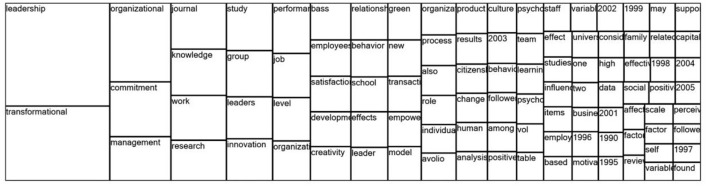
Treemap.

The treemap is a diagram presenting hierarchical segments into a set of nested rectangles of different values and sizes. The reader can use a rectangular size to understand the number of coding in each node. A treemap was used to verify the best-defined area in rectangular form and to link the factors. The larger areas are presented on the left side and the smaller rectangular areas are presented on the right side (as shown in Figure 4). The words “leadership” and “transformational” were mostly used in the studies on the public universities of Saudi Arabia and the weightage of the words was 2.39 and 1.73%, respectively. However, organizational commitment and knowledge management were the second-highest practices in the public universities, and the weightage of the words was 0.75 and 0.60%, respectively, etc. Therefore, the reader can easily imagine the values counted for the rectangular areas in the treemap and the weightage percentage.

**Figure 5:**
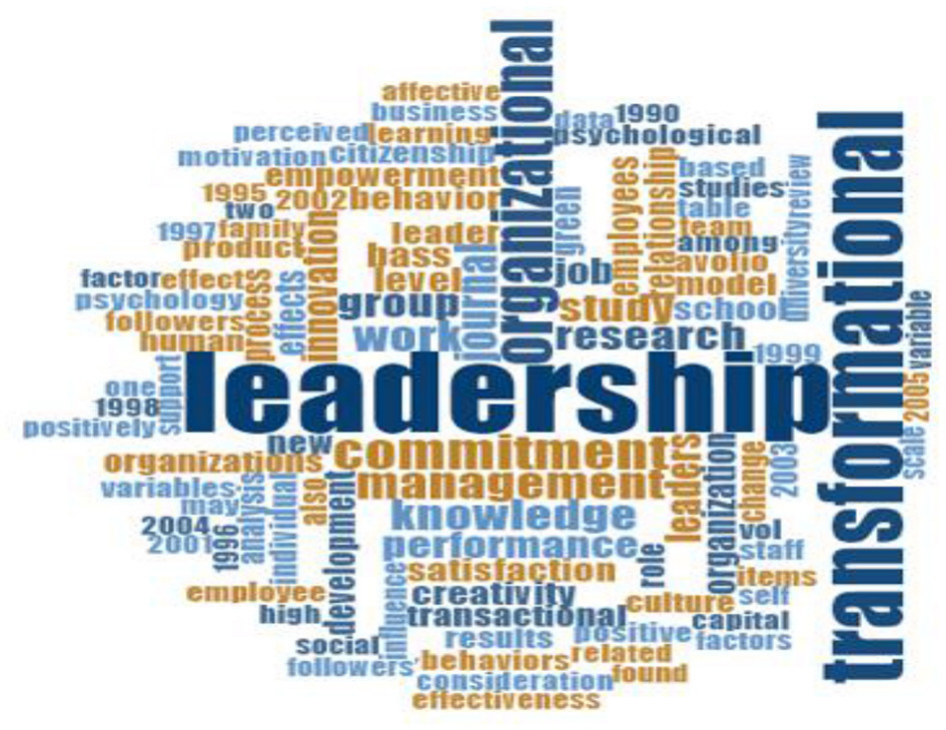
Word cloud query.

This study presents the results of the word cloud by applying the technique of word frequency query (Figure 5). The word cloud signifies the words in the study which were mostly used by the researchers and social scientists in their studies. It highlights the words “transformational leadership” which was mostly applied in the studies on public universities. Second, the organizational commitment was the second-highest base of the public universities in Saudi Arabia.

### Mind Map

**Figure 6:**
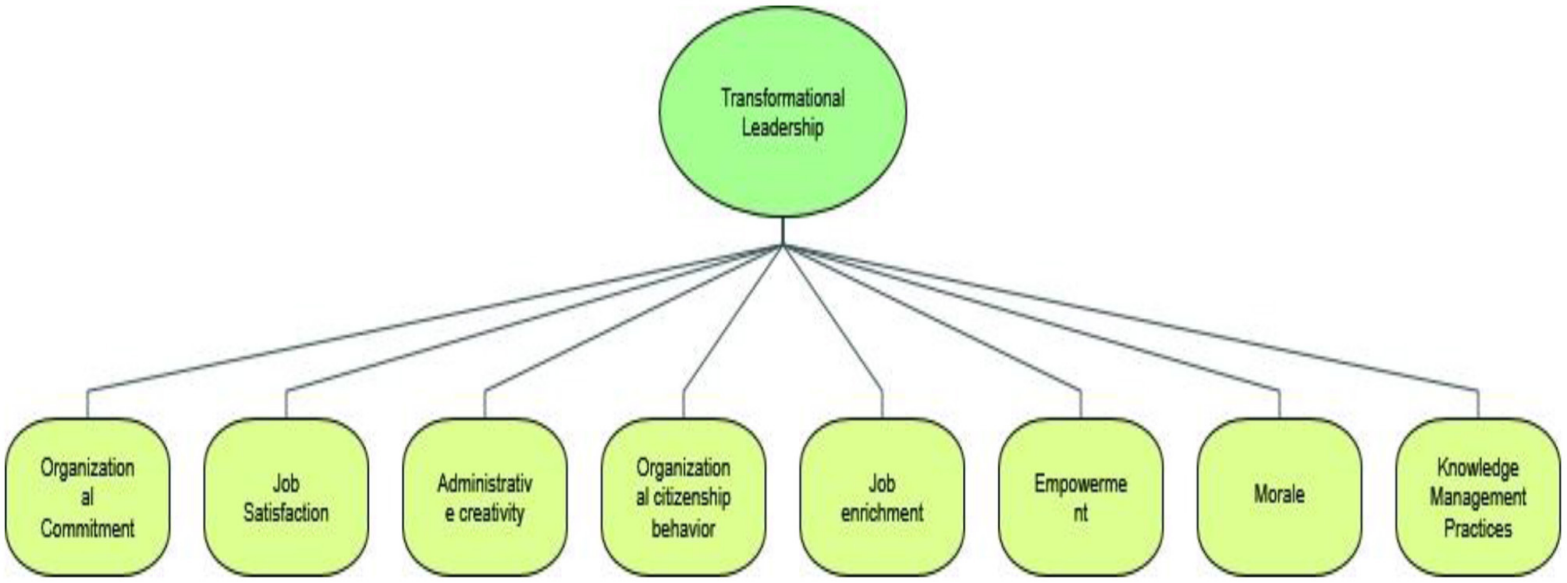
Mind map.

A mind map was used to brainstorm the ideas of the researchers defined in the studies. The mind map depicts the thinking level from a single theme and is casually presented immediately and spontaneously. Mind maps can be used to explore the related linkages between transformational leadership and organizational outcomes such as organizational commitment, job satisfaction, administrative creativity, organizational citizenship behavior, job enrichment, individual empowerment, morale, and knowledge management practices (Figure 6). Therefore, the main thematic base is transformational leadership that explores the multiple sub-themes including the parent nodes and child nodes. This study also presents the text search queries (as shown in Appendixes).

## Discussion

Administrative leadership has been greatly affected by global changes at both organizational and national levels. Therefore, the need for an innovative leader who can lead the development and adapt to various changes in the globalized and continuously changing world has become vital in government and private organizations, particularly as it relates to a leadership capable of managing transformations and transforming visions into action. Interestingly, transformational leadership behavior has become a basic requirement for all organizations that aim for continuity and excellence. Therefore, instilling transformational leadership can now be viewed as a prerequisite for managers at various administrative levels. This approach aids in the development and adaptation of different environmental challenges, while also motivating the followers to achieve the goals of their organizations effectively and efficiently.

As shown in this study, it has been established that transformational leadership has certain dimensions and characteristics, which may be personal or those that deal with others within an organization or its surrounding environment. These characteristics, whenever found in the leaders of an organization, give a strong indication of how effectively an organization is approaching its goal, maintaining development, and dealing with internal and external environmental challenges. This is what government and private organizations alike need to transform into advanced organizations that adapt to changing environments in all dimensions, may they be social, economic, cultural, political, informational, or technological. The results show 22 studies that investigated transformational leadership practices in Saudi Arabian public universities. The levels of practice of the four aforementioned dimensions of transformational leadership found in the sample ranged between two levels. Interestingly, the organizational commitment was found to have the highest leverage (9 studies out of 22 of the conducted research in the public Universities of Saudi Arabia). It shows that the researchers practiced transformational leadership to assess the organizational commitment (emotional attachment) of the faculty members in Saudi Arabian Universities (Dubinsky et al., 1995; Bruch and Walter, 2007; Tengi et al., 2017; Purnomo and Novalia, 2018). Furthermore, organizational commitment was proven to be the most critical and researched behavior in Saudi Arabian Universities (Al Ubiri, 2016).

Out of 22, seven studies found that transformational leadership had a significant contribution to individual empowerment (Alsomaly and Metoly, unpublished). It means that empowerment is the second highest and critical behavior of the individuals in Saudi Arabian public universities such that the researchers have examined it in latest two decades. Out of 22, five studies have been found concerning the linkages between transformational leadership and both job satisfaction (Penger and Cerne, 2014) and knowledge management practices (Tuan, 2013). This means that job satisfaction and knowledge management practices were parallel and offered the same opportunity for researchers to explore them by using transformational leaders. Additionally, out of 22, four studies were conducted on the linkages between transformational leadership and both organizational citizenship behavior (Al Ubiri, 2016) and administrative creativity (Ayyad, 2013; Al Rashidi, 2017; Atoum, 2018). This shows that in terms of the importance of the predicted variables, these behavioral factors were the least important in Saudi Arabian Universities and were quite different from the actual behavioral tendencies (Alsomaly and Metoly, unpublished). The least important behavioral tendencies such as morale and job enrichment have been studied in the context of Saudi Arabian Universities by hiring transformational leaders.

The findings of the studies asserted the importance of transformational leadership practice as a leadership method in government universities due to its characteristics that are most suitable for the development of the universities, and its multiple positive effects on the organizational structure of universities, the behavior of their employees, their job performance, the quality of academic research, and community service outcomes. The results are consistent with the studies: each dimension of transformational leadership significantly contributes to the actual performance of the academic educational institutions because all dimensions have motivational and cognitive abilities which are considered necessary for the development and assessment of academic performance (Al Gabri, 2018); transformational leadership in Saudi Arabian public institutions promotes higher levels of staff opinions and generates higher academic performance (Al Amiri, 2002); it has the best practices in developing and generating novel ideas that enable the process of knowledge sharing in terms of knowledge management (Al Madhahaji, 2017); it is the best practice to satisfy the employees at a workplace and in turn, the employees do more to compensate their goodwill so there is a higher job satisfaction in educational institutions (Al Mandil and Shawi, 2018) as well as in Saudi Arabian technical educational institutions (Al Miman, 2013); it has an influential effect on organizational creativity in terms of knowledge sharing practices, developing novel ideas, and doing best practices and processes in private and public higher institutions in Riyadh (Al Rashidi, 2017); it enables corporate social responsibility among the faculty members of King Saud University (Al Regeb, 2017); it helps to empower the knowledge management system in Saudi Arabian public universities (Al Saleh, 2019); and it is the ultimate antecedent of both organizational commitment and organizational citizenship behavior (Al Ubiri, 2016) because it enhances faculty commitment and citizenship behaviors among the faculty members of public higher institutions. The results also showed that the level of transformational leadership practice in Saudi universities is mostly high and that several recommendations were made to enhance the practice of this leadership method in a way that helps improve the government universities, enabling them to effectively achieve their goals. These recommendations can be summarized as follows:

- The necessity of providing an appropriate supportive environment for university leaders to practice transformational leadership in its various dimensions and to motivate, encourage, and empower leaders at all organizational levels in the interest of change and development. It is also important to develop and nurture the skills and leadership characteristics of the members of an organization through material and moral incentives. It can be seen that the personal characteristics and individual capabilities that exist in academic leaders create an encouraging environment that fosters the practice of transformational leadership while developing academic departments, influencing the performance of their fellow faculty members, and building new cohorts of leaders (Al Gabri, 2018; Al Shammari, 2020; Al Zahrani, 2020).
  It is important to develop the generalized and transformational thinking skills of academic leaders, along with providing specialized training programs and mandatory workshops on the various dimensions, skills, and behaviors of transformational leadership for university leaders, using the best global practices and programs in which the leaders from the different universities can exchange experiences (Ayyad, 2013; Burqan, 2013; Al-Shammari, 2015; Al Madhahaji, 2017; Al Rashidi, 2017; Al Gabri, 2018; Al Saleh, 2019; Al Zahrani, 2019; Al Shammari, 2020).
- Considering the characteristics of transformational leadership as an important trait when appointing new academic leaders at universities, and setting specific measures by which people who have transformational leadership credentials are chosen. Accordingly, those individuals must have inspirational personalities that stimulate and motivate the employees and ultimately, become efficient leaders capable of creating a clear future vision. This highlights the importance of establishing clear criteria for selecting leaders in universities and involving experienced faculty members in the process of selecting academic leaders (Ayyad, 2013; Al Ubiri, 2016; Al Sharari, 2017; Al Mandil and Shawi, 2018; Al Saleh, 2019; Wazrah, 2019).
- Activating the role of academic leaders in transforming knowledge into a competitive advantage by employing the dimensions of transformational leadership with its positive impact on knowledge sharing and organizational learning. Focusing on their role in elevating the trends of knowledge sharing, exercising idealized influence, motivating faculty members to share their knowledge and experience, providing intellectual stimulation for more creativity and innovation, and supporting them to excel in administrative and academic work and training (Al Madhahaji, 2017; Al Zahrani, 2019).
- University leaders must exhibit transparency and justice in all administrative aspects and decisions while reaching high levels of proficiency and discipline in work. This is achieved through participation in decision-making and understanding the implications of those decisions on the workers. The leaders must be role models in their behavior and discipline at work in order for the workers to learn these values. Universities must ensure that academic leaders lead by example and exhibit exemplar behaviors, practices, and decisions that serve the public interest (Al Regeb, 2017; Al Aklibi, 2019; Al Zahrani, 2020).
- Leaders must appreciate the efforts of employees, encourage and inspire them to succeed, and give them enough freedom to find new solutions to existing problems. This kind of approach leads to success in creative thinking and employee empowerment. It is also necessary that the transformational leadership methodology and administrative creativity are in harmony with the strategic goals of universities (Al Subaie, 2013; Atoum, 2018; Somali and Motwali, 2018).
- A supportive organizational environment for transformational leaders must be established in Saudi Arabian universities to attract creative minds. More work is needed to conduct meetings and scientific seminars that promote the application of the transformational leadership dimensions by university leaders, and work to create an organizational culture that is consistent with the requirements of transformational leadership, encouraging creative ideas, team spirit, accountability, and exemplary work values (Al Regeb, 2017; Al Rashidi, 2017; Al Aklibi, 2019).
- Finally, it is recommended that effort be put into measuring the level of job satisfaction and the morale of organization members while promoting the practice of transformational leadership among leaders. Such an approach is considered as one of the factors that help in raising the levels of job satisfaction and morale among faculty and administrators, while also helping universities to achieve their goals (Al Miman, 2013; Al Mandil and Shawi, 2018; Al Zahrani, 2020).

## Conclusion

Organizations need transformational leaders who believe in the culture of change and development and seek such a culture with sincerity and determination. The characteristics of the leader and the application of the transformational leadership dimensions are positively correlated with the commitment of the followers to achieve goals in a framework of trust, shared values, and a shared vision. The dimensions can be summarized in the following points:

(a) Leaders must have a clear vision and mission, consistent values, high standards, and a clear sense of purpose, confidence in self and others, and the ability to gain the trust and respect of followers. They must also demonstrate the importance of the human element in development and progress by paying attention to the needs of individuals, harnessing the potentials of said individuals, and aiding individuals in self-improvement and achievement.
(b) Leaders must pursue new ideas and approaches to getting a job done effectively. This entails influencing the motivation and enthusiasm of the team with charisma, exhibiting exemplary behavior, inspiring, and providing creative encouragement and intellectual stimulation.
(c) Leaders must demonstrate individual traits, such as the ability to focus, pay attention, change, and take risks by establishing consistency between words and actions. They must also set an example in handling workloads, and demonstrate the ability to communicate and reach out to others by showing empathy and maintaining a high degree of harmony and cooperation between the members of the group to raise the morale among the members.

Following a review of both theoretical and empirical research in the field, this study has found that this pattern of administrative leadership, “transformational leadership,” has multiple positive effects on individuals and the organizations for which they work. Accordingly, transformational leaders can motivate their followers to develop feelings of acceptance, take greater ownership of their work, and transfer their personal interests to a collective interest that is integrated into the interests of their organizations, leading to higher levels of organizational commitment. Adopting transformational leadership techniques also allows for integration, harmony, learning, and innovation in human resource management. Transformational leadership positively affects organizational health and can be used as an intervention technique to improve the organizational environment, ultimately affecting performance levels.

Transformational leadership has a positive impact on many behavioral variables, including job satisfaction among organization members, the sense of organizational justice among followers, organizational citizenship behavior, follower motivations, and emotional commitment to change and to the organization (i.e., facing the negative consequences of work stress, creating a positive work environment, and spreading optimism). In addition, the transformational leadership style is teachable. As such, it is possible to hold training courses for followers at various organizational levels to deepen their awareness of the concept of transformational leadership behavior to help foster the rapid internalization of such ideals in various organizations. This could lead to performance improvement at the organizational level, preparing firms better to confront new challenges head-on.

## Data Availability Statement

The original contributions presented in the study are included in the article/Supplementary Material, further inquiries can be directed to the corresponding author.

## Author Contributions

All authors listed have made a substantial, direct and intellectual contribution to the work, and approved it for publication.

## Conflict of Interest

The author declares that the research was conducted in the absence of any commercial or financial relationships that could be construed as a potential conflict of interest.

## Publisher's Note

All claims expressed in this article are solely those of the authors and do not necessarily represent those of their affiliated organizations, or those of the publisher, the editors and the reviewers. Any product that may be evaluated in this article, or claim that may be made by its manufacturer, is not guaranteed or endorsed by the publisher.
