# Supplementary material for: The Dimensions of Transformational Leadership and Its Organizational Effects in Public Universities in Saudi Arabia: A Systematic Review
Source: Front Psychol. 2021 Nov 10;12:682092. doi: 10.3389/fpsyg.2021.682092 (PMC8635992; doi:10.3389/fpsyg.2021.682092)
Supplement: Supplementary file 1 [file Data_Sheet_1.pdf]

Appendixes

Words weightages

Word	Length	Count	Weighted Percentage (%)
leadership	10	3295	2.39
transformational	16	2387	1.73
organizational	14	1487	1.08
commitment	10	1027	0.75
management	10	827	0.60
journal	7	787	0.57
knowledge	9	775	0.56
work	4	761	0.55
research	8	684	0.50
study	5	669	0.49
group	5	659	0.48
leaders	7	596	0.43

Text search Query

Transformational Leadership

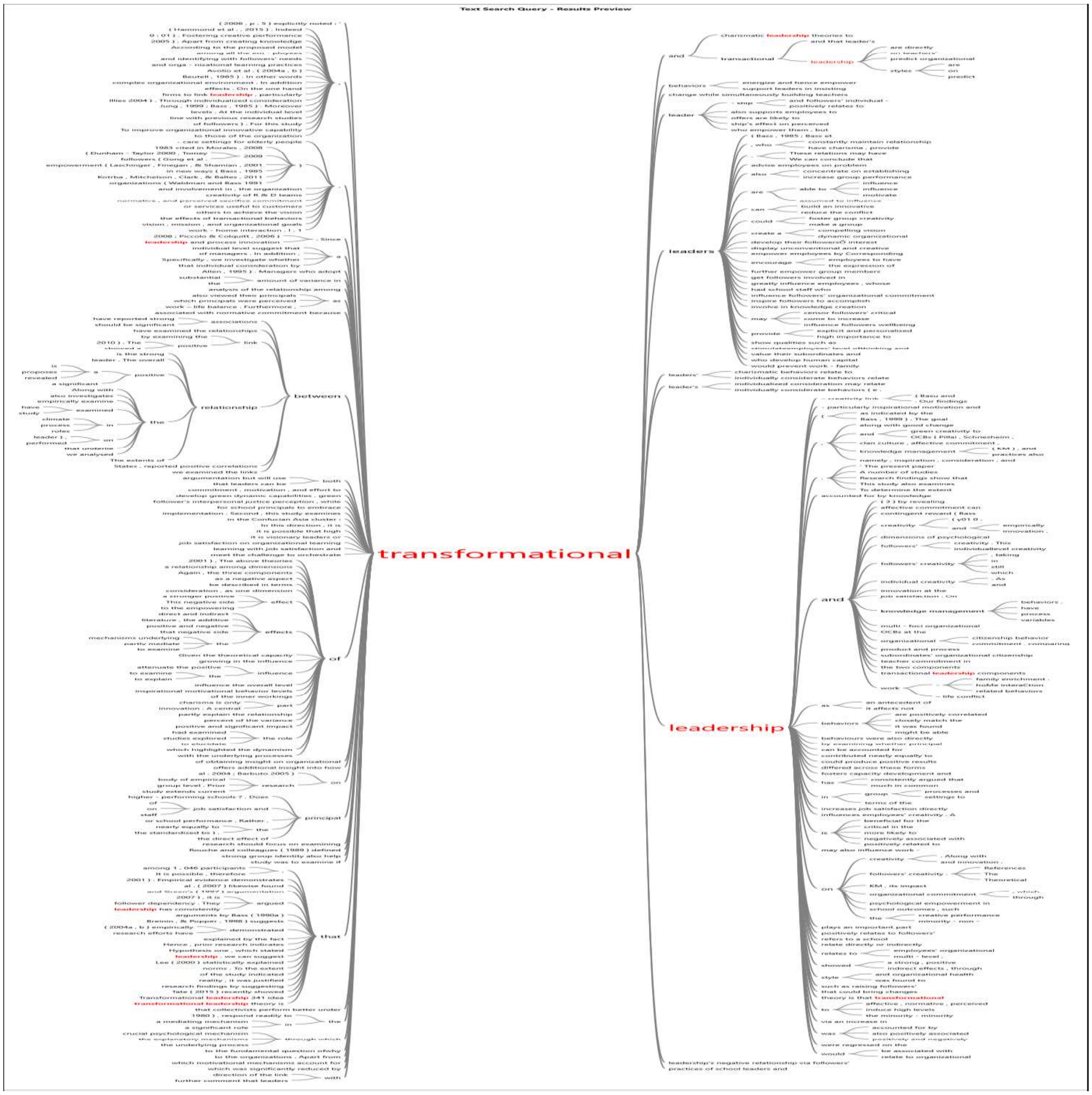

Morale

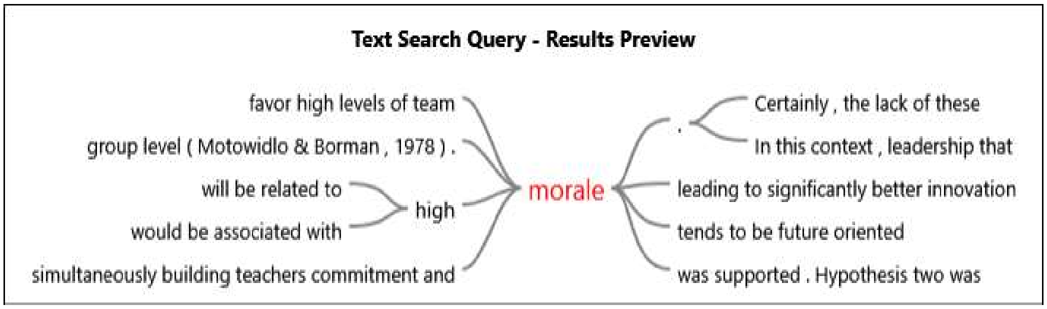

Individual empowerment

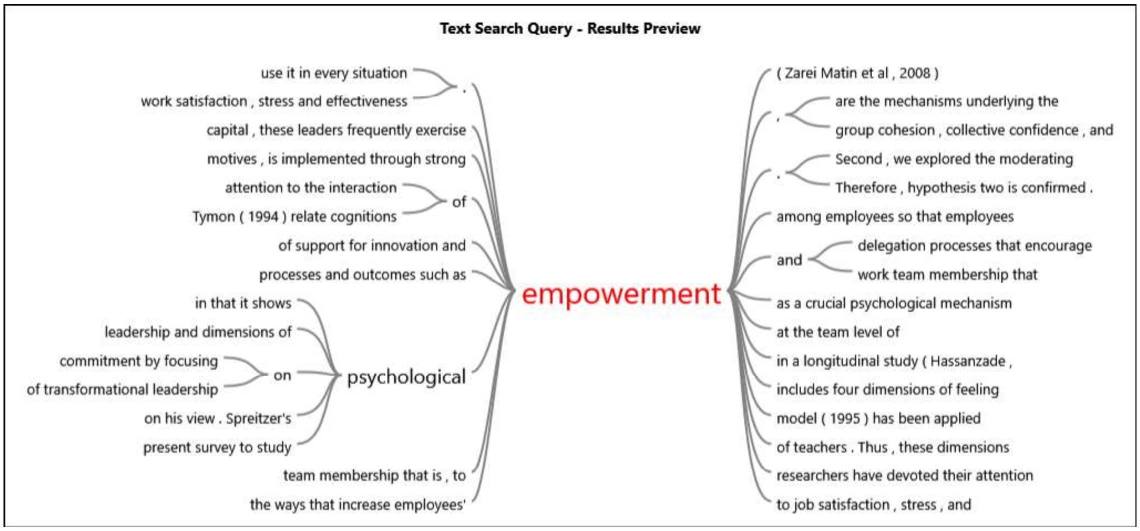

Job satisfaction

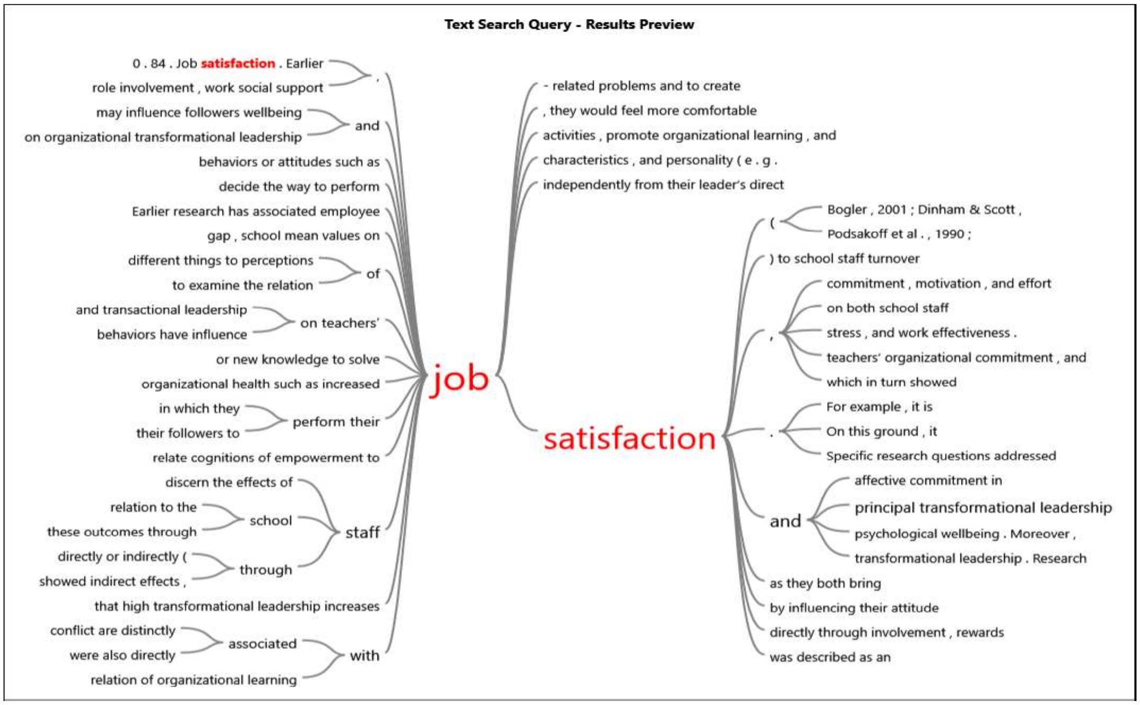

Administrative creativity

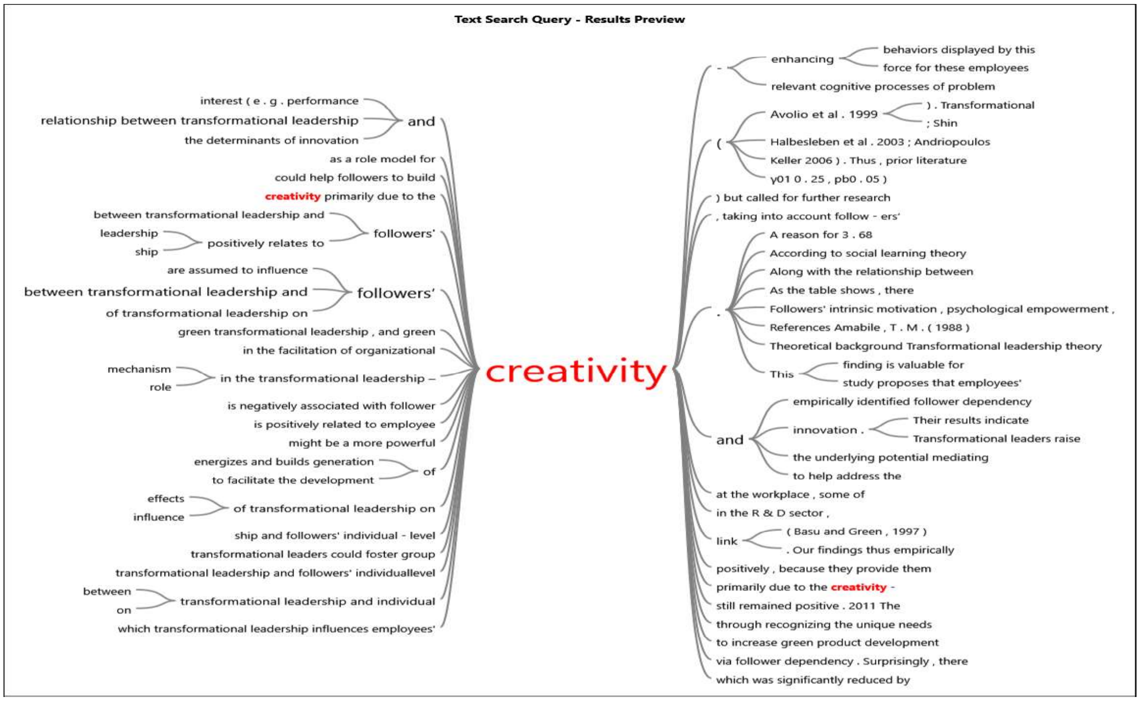

Knowledge management practices

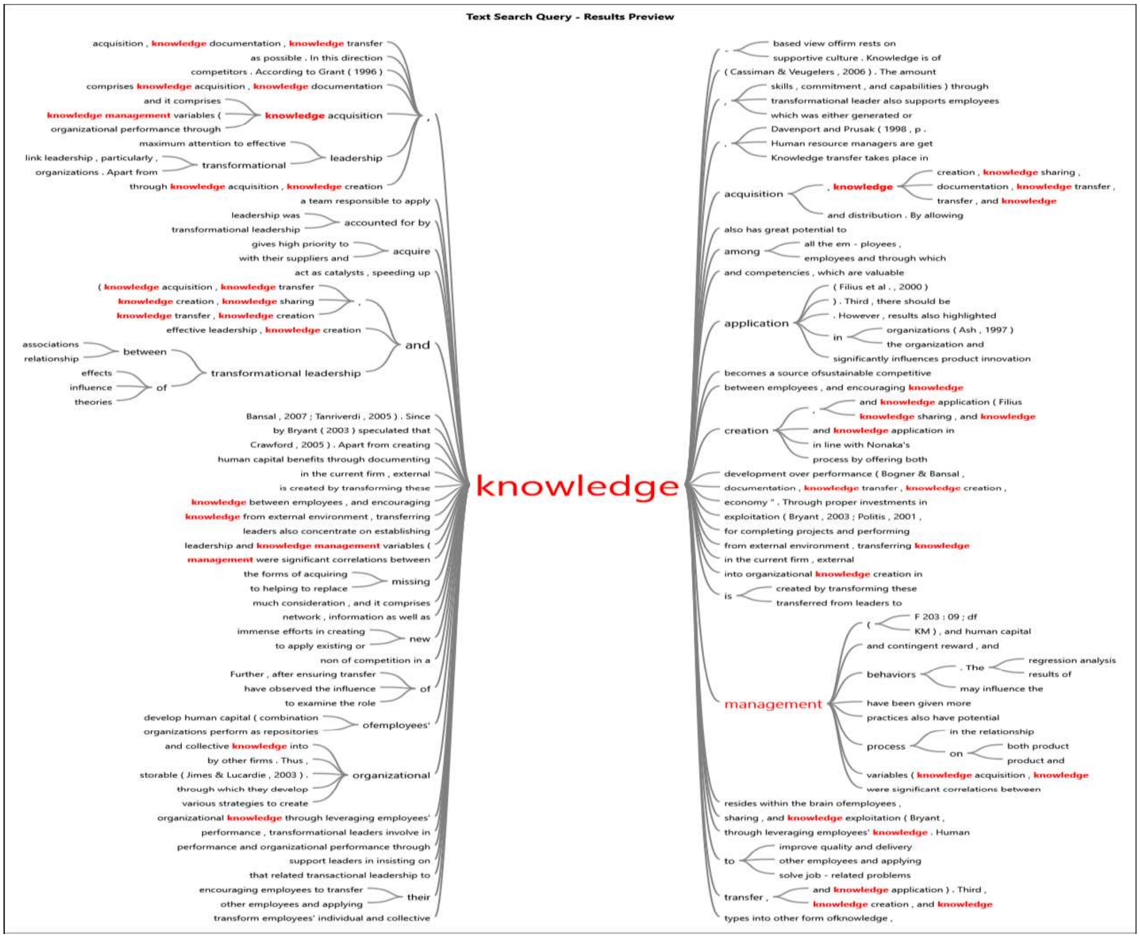

Organizational commitment

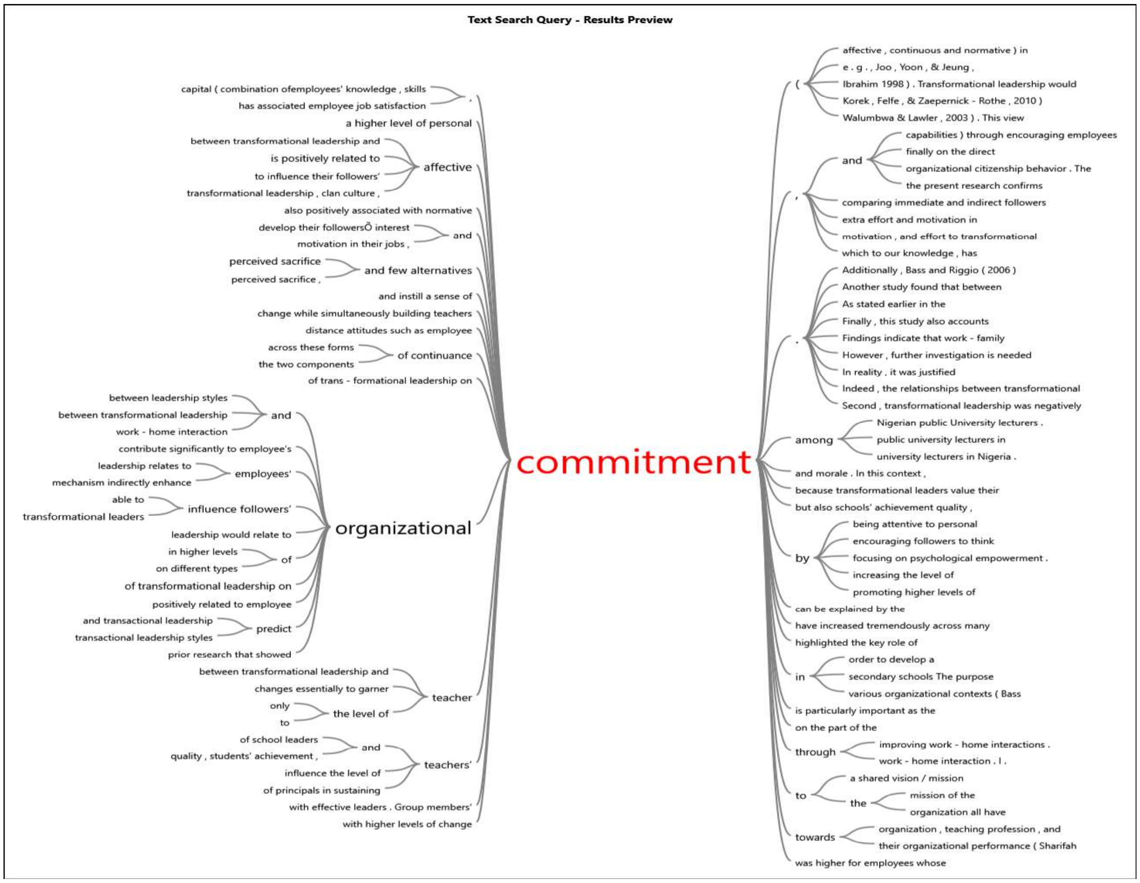

Organizational citizenship behavior

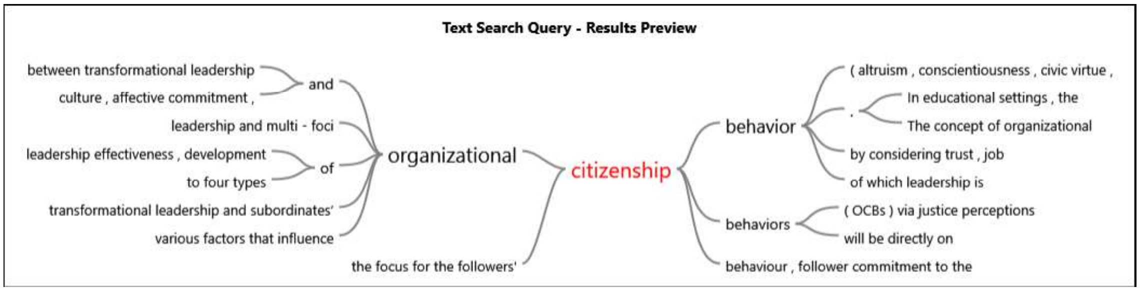

Job enrichment

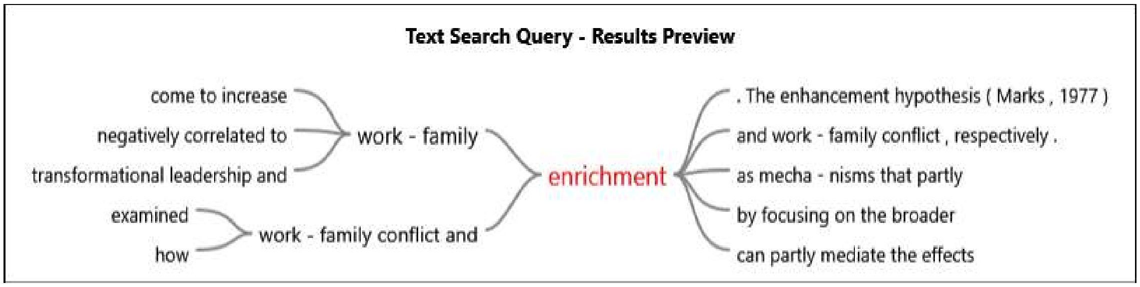

Project Map

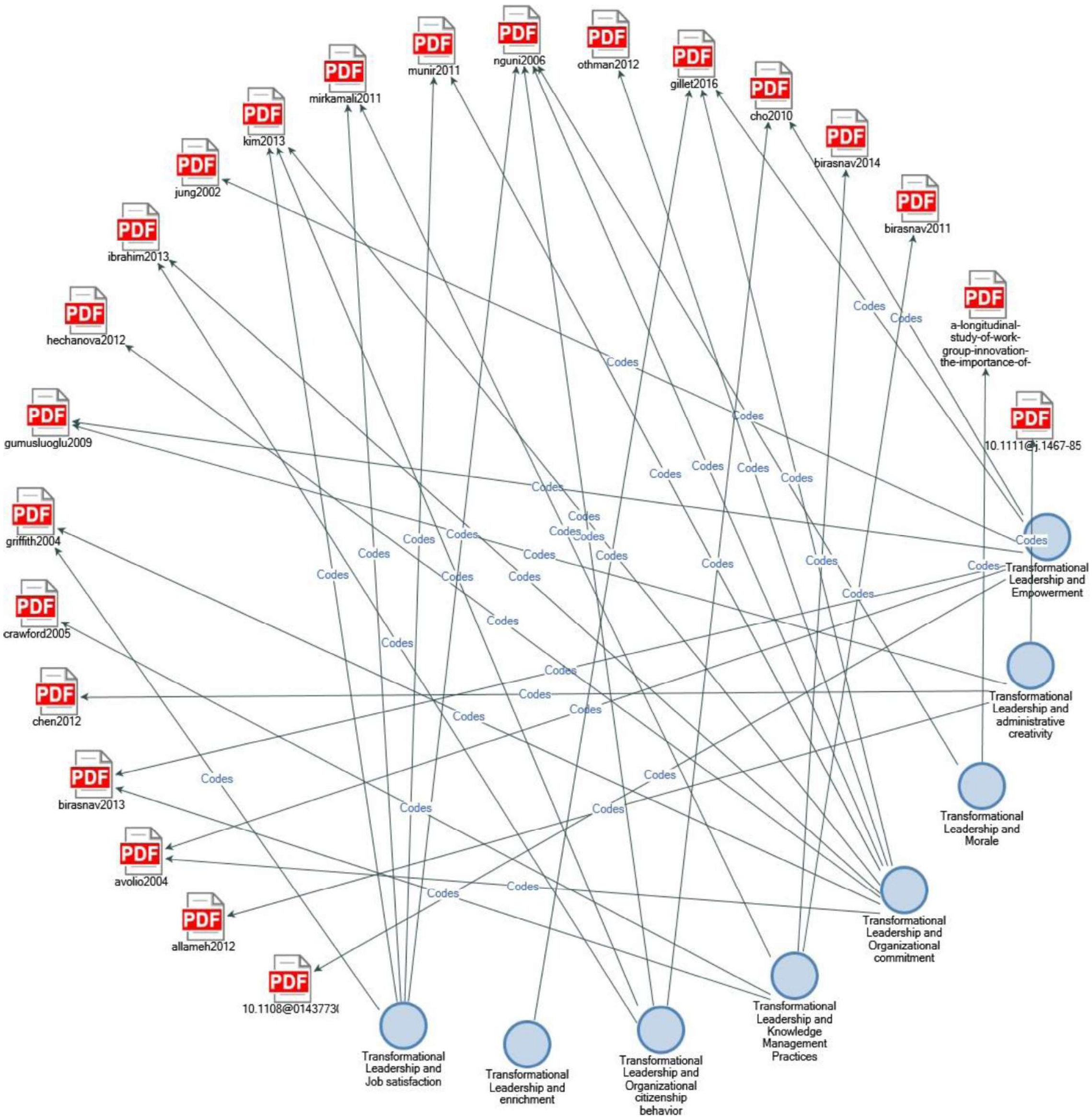

munir2011
nguni2006
othman2012
mirkamali2011
gillet2016
cho2010
kim2013
birasnav2014
jung2002
birasnav2011
ibrahim2013
hechanova2012
a-longitudinal-study-of-work-group-innovation-the-importance-of-
gumusluoglu2009
10.1111@j.1467-85
griffith2004
Transformational Leadership and Empowerment
crawford2005
chen2012
Transformational Leadership and administrative creativity
birasnav2013
Transformational Leadership and Morale
avolio2004
allameh2012
Transformational Leadership and Organizational commitment
10.1108@0143773(
Transformational Leadership and Knowledge Management Practices
Transformational Leadership and Job satisfaction
Transformational Leadership and enrichment
Transformational Leadership and Organizational citizenship behavior
Codes
